# Assessment of the Dynamics of Temperature Changes in the Knee Joint Area in Response to Selected Cooling Agents in Thermographic Tests

**DOI:** 10.3390/ijerph18105326

**Published:** 2021-05-17

**Authors:** Aleksandra Radecka, Waldemar Pluta, Anna Lubkowska

**Affiliations:** Department of Functional Diagnostics and Physical Medicine, Pomeranian Medical University in Szczecin, Żołnierska 54 Str., 71-210 Szczecin, Poland; waldemar.pluta@pum.edu.pl (W.P.); anna.lubkowska@pum.edu.pl (A.L.)

**Keywords:** local cryotherapy, thermal imaging, cooling agents, liquid nitrogen vapors, cold air, ice bag, thermography

## Abstract

Although local cryotherapy (LC) is performed with various cooling agents (C_Ag_) such as ice, water, and gasses, in clinical practice, it is mostly performed with cooling gasses. Presently, LC with cooling gasses is very popular but the inference about the thermal (stimulus) effect on the tissues is mainly based on research carried out using ice packs. The proposed objective of the study was to evaluate the dynamics of temperature changes in the knee joint area in response to a 3-min exposure to liquid nitrogen vapors (LNVs), cold air (CA) and ice bag (IB). The study group included 23 healthy volunteers with an average age of 26.67 ± 4.56. The exposed (ROI_E_) and contralateral (ROI_NE_) areas of the knee joint after exposure to C_Ag_ were observed. Immediately after 3 min of LC, the ROI_E_ temperature dropped by 10.11 ± 0.91 °C after LNV, 7.59 ± 0.14 °C after IB and 6.76 ± 1.3 °C after CA. Significant tissue cooling was maintained up to 15 min after LNV (*p* < 0.01), 10 min after IB (*p* < 0.05) and 5 min after CA (*p* < 0.05). LC causes significant temperature changes both in ROI_E_ and ROI_NE_. The greatest cooling potential was demonstrated for LNV and the lowest for CA.

## 1. Introduction

Cryotherapy is a physical procedure involving the use of a cooling agent (C_Ag_) to temporarily disturb thermal homeostasis. In response to the tissue cooling, a number of thermoregulatory reactions take place that make up the post-treatment reaction, being induced to achieve a therapeutic effect [1,2,3]. The treatment performed on healthy people (e.g., in biological regeneration) for stimulation purposes is named cryostimulation [4]. The therapeutic effects of cryotherapy include, among others: analgesic, anti-inflammatory, anti-swelling, myorelaxant, hyperemic, and modulating antioxidant and immune responses of the organism [1,5,6,7,8,9]. On healthy people, e.g., in athletes, cryostimulations are performed in order to improve muscular recovery after exercise [10,11,12]. The therapeutic or stimulating effect of cryotherapy depends on the ability to reduce the tissues’ temperature, but the degree of hypothermia has not been clearly defined in the literature.

Cryotherapy treatments are divided into whole-body cryotherapy (WBC), which are performed in a cryochamber with a whole-body exposure, partial-body cryotherapy (PBC) performed without exposure of the head, and local cryotherapy (LC) [2,4,13,14], where a small area of the body is exposed to low temperatures [15,16]. Thanks to mobile technologies of vaporizers, LC can be carried out by using refrigerant gases, for example, liquid nitrogen vapors (LNVs). In over the past several years, LC has become a trendy treatment, with LC useable directly over the regions of interest (ROIs) by athletes and teams during sport events [17]. Nowadays the term local cryotherapy is often understood very broadly, covering the use of various cooling agents, various treatment procedures, also including the duration of exposure. Consequently, in publications there are descriptions of the LC methodology in which both various forms of cooling compresses (ice bag (IB), frozen peas, ice towels, ice massage, gel packs, hypothermic blanket) and refrigerant gases (cold air (CA), carbon dioxide microcrystals, liquid nitrogen vapors) [5,7,8,9,10,18,19,20,21,22,23,24,25,26,27,28,29,30], and cryotherapy units (that can provide a continuous or intermittent circulation of ice water from an insulated container to a pliable cooling pad placed onto the treatment area) [31,32].

In reports describing the effect of using LC, the number of exposures ranges from 1 to 20, the temperature of C_Ag_, ranges from 0 °C to −160 °C and the duration of application ranges from 2 to 30 min [8,9,21,22,23]. Despite such a wide variety of LC treatments (methodology, cooling factors), no exhaustive verification of the physiological thermal response (degree and duration of tissue cooling) depending on the parameters used was not carried out. All LC procedures are assigned the same effects, which seems to be a simplification or even a mistake. Due to the different physical properties of the mentioned C_Ag_ (temperature, physical state, thermal conductivity), the dynamics and intensity of thermal changes induced in the exposed tissues (especially skin and subcutaneous tissue) may be varied, and the therapeutic effects may differ significantly, which in turn causes interpretative discrepancies in the literature on the subject [1,33]. In extreme cases, failure to take into account the physical properties and failure to adapt methodological principles to the application of a selected C_Ag_ (e.g., direct contact with the skin, prolonged duration of exposure) may result in frostbite, burns, and even nerve damage and slower wound healing [8]. Additionally, as rightly noted by Massimo De Nardi et al., despite the proliferation of scientific reports on these cooling technologies, there is still a lack of information concerning their effective benefits related to the optimal exposure protocols and the relationship with the treatments’ supposed effects [17]. In our opinion, this is especially true of the local use of refrigerant gases in LC, although LC induced by vaporizing gasses is very common in wellness centers and cryotherapy facilities globally, we only found a few studies which investigated the thermal effects after these cooling agents. The physiological response directly depends on the effectiveness and maintenance time of tissue cooling (cooling potential). It seems to be important that, before introducing LC with cooling gases into patient therapy, one should thoroughly understand the physiological thermal reaction to the stimulus.

Skin temperature is a very important physical attribute used as a diagnostic parameter in medicine and sport [6,34]. Changes of skin surface temperature indicate, among other things, changes in the peripheral blood flow and may be the basis for the assessment of post-treatment reaction as well as post-traumatic or inflammatory changes [34]. The method allowing for detailed registration of these changes is, among others, thermography. This technique uses cameras that detect and record radiation, invisible to the human eye, emitted by the human body in the wavelength range of 8–12 μm. The collected data, after using specialized software, enable the generation of an image (thermogram/thermograph) showing the temperature of the examined area [35]. Important advantages of the thermographic method are complete safety, non-contact and non-invasive nature of measurement with the simultaneous possibility of thermographic mapping of the distribution of skin surface temperature [36]. Thermographic measurements are used, among others, in: the prevention and treatment of sports injuries, detection of delayed muscle soreness, evaluation of brown adipose tissue activation, evaluation of cryotherapy protocols, or the evaluation of the effectiveness of other physiotherapeutic treatments [1,6,34,37,38,39,40]. The literature reports that thermography is a frequently used and proven method of measuring body temperature after cryotherapy [41,42,43]. Thermographic evaluation can be conducted in real time, providing information on the strength, efficiency and extent of the stimulating activity. Ultimately, it is the basis for the subsequent interpretation of the correctness and effectiveness of the applied procedure and understanding of the phenomena caused by the applied stimulus.

There are several studies on the effect of cooling with cooling gases on tissue temperature, but these were carried out using the WBC and PBC procedures [3,44,45]. Even attempts have been made to the theoretical modeling of time-dependent skin temperature, but also during WBC [46]. However, due to the extremely different area of exposed tissues, it cannot be compared with LC. Single studies evaluate the cooling potential of gaseous refrigerants used in the LC, but many of them are published in the non-English literature, which significantly limits their range [47,48,49,50]. Presently, clinicians are exposing individuals to extreme temperatures (cooling gas temperature ranging from −40 to −190 °C) based on anecdotal evidence and very little is known regarding its effectiveness or the physiological changes that occur during or after the treatment. In addition, the question arises whether the use of cooling gases is necessary in LC. Perhaps a similar physiological thermal reaction effect can be available after a short-term use of well-known and widely described compresses with ice.

Precise knowledge of the organism’s response to the stimulating activity of individual C_Ag_ is crucial in planning their inclusion in the therapeutic process. The literature still lacks detailed data regarding the influence of the type of C_Ag_ used and the duration of exposure in LC treatments on the organism’s response. The above were implications for undertaking research aimed at assessing the dynamics of changes in temperature in the knee joint area in response to a 3-min exposure of the knee joint to LNV, CA and IB. The change in skin temperature after LC was recorded using the thermographic method. The most commonly used clinical C_Ag_ were used in the study at the procedurally recommended time of 3 min for gas blows [51]. Although the literature reports a very different duration of exposure to IB, we decided to standardize it while planning the study in order to compare the cooling potential of the agents used, focusing on the observation of changes in the surface temperature of the anterior area of both knee joints—stimulated and contralateral.

## 2. Materials and Methods

The research was approved by the Bioethics Committee of the Pomeranian Medical University (Ref. No.: KB-0012/36/13). The research was conducted at the Research Center for Impact of Cryogenic Temperatures on the Human Body (Chair and Department of Functional Diagnostics and Physical Medicine, Pomeranian Medical University in Szczecin) in the period from July to September 2020. The research was financed by a grant from the Ministry of Science and Higher Education obtained by the Faculty of Health Sciences of the Pomeranian Medical University in Szczecin [WNoZ-318-01/S/13] [6570/IA/SP/2016].

### 2.1. Volunteers

Healthy individuals aged 20 to 40 were recruited for the study. During the recruitment period, 31 volunteers entered the study who underwent medical verification in terms of their health condition. Volunteers in the questionnaire survey were asked about medications taken and infectious diseases in the last 4 weeks preceding the study, and the women additionally about their menstrual cycle. The exclusion criteria for the study were as follows: chronic cardiovascular diseases, metabolic diseases, inflammatory diseases of the musculoskeletal system and previous injuries within the lower limbs in the last 2 years, current medications taken, ongoing infections, ongoing menstruation in women. Ultimately, a group of 11 women and 12 men was included in the study, healthy, without presence of chronic cardiovascular diseases, metabolic diseases, inflammatory diseases of the musculoskeletal system and previous injuries within the lower limbs in the last 2 years. Volunteers were informed in detail about the purpose and course of the planned research procedures.

**Figure 1:**
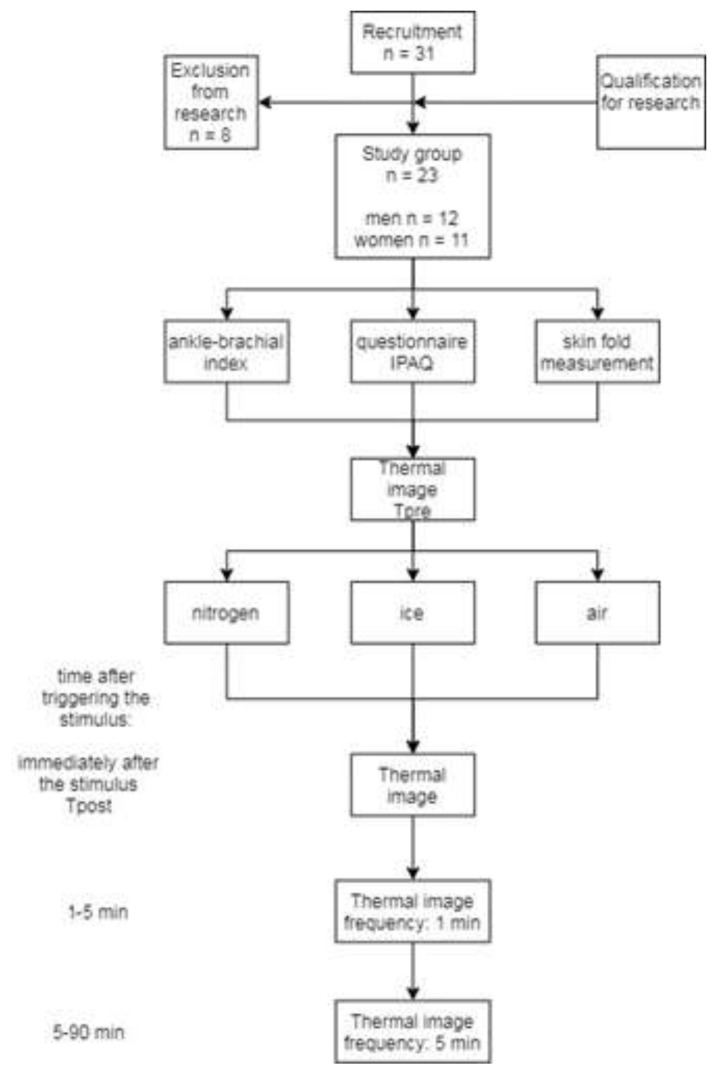
The course and sequence of research procedures.

All qualified volunteers gave their written consent to participate in the research, in line with the Helsinki Declaration. Detailed characteristics of the study group are presented in Table 1, while the course and sequence of research procedures are presented schematically in Figure 1.

### 2.2. Research Procedures

The study was conducted in the system of triple participation of the subjects (a different C_Ag_ was used during each meeting) at intervals of at least 4 days (to eliminate the summation of thermal effects and the risk of inducing adaptation). The order of selection of the C_Ag_ used was random and the volunteer was not informed about the name of the C_Ag_ used. The volunteers were instructed in detail on how to prepare for the test in accordance with the Thermographic Imaging in Sports and Exercise Medicine (TISEM) guidelines [40].

During the first meeting, the level of physical activity was assessed using the Polish version of the International Physical Activity Questionnaire (IPAQ), which characterizes daily physical activity in MET-min/week [52]. The thickness of the supragenual skinfold was also measured at a distance of 3 cm from the base of the patella using a skinfold caliper (Skinfold Calliper, Harpenden, England), accurately calibrated and with a constant spring pressure of 10 g/mm^2^ throughout its entire range (measuring range: 0 mm to 80 mm, accuracy: 99.00%).

During each meeting, before performing the LC procedure, the hemodynamic parameters were assessed, which included the measurement of blood pressure, heart rate and the ankle–brachial index (ABI) values for the right and left lower limbs (oscillometric method, MESI ABPI MD apparatus). The volunteer was rested in a supine position 15 min before and during the measurement of hemodynamic indices.

### 2.3. Thermal Imaging

According to the standards of thermal imaging tests, before taking the first thermal image, the subjects rested for 15 min with their lower limbs uncovered (elimination of the clothing effect and thermal acclimation). Subsequently, thermographic imaging of the lower limbs and the LC procedure were performed, using one of the three cooling agents, according to the procedure described below. The application of C_Ag_ in all subjects and each time was performed in a sitting position for 3 min, only in the area of the anterior surface of the left knee joint. Immediately after the end of the treatment, thermographic measurements were made (according to Figure 1) for a period of 90 min. From the beginning to the end of the research procedures, the volunteer sat on the edge of the chair, touching only its surface with his/her buttocks. At the moment of the thermographic measurement, the participant stood up.

During the measurement, the volunteer was in a relaxed standing position with the feet parallel to each other and hip-width apart. During taking thermal images, the camera was placed on a tripod perpendicular to the anterior area of both knee joints. Each time, the camera was turned on at least 10 min before the T_0_ measurement and immediately before each subsequent measurement it was calibrated in order to obtain the highest possible sharpness of the images. The temperature and relative air humidity in the room were recorded using a thermohygrometer (digital thermohygrometer, TFA Dostmann, Wertheim-Reicholzheim, Germany) and taken into account when configuring the thermal imaging camera. Thermograms were taken in a room with a humidity of 50% and a temperature of 23 ± 1 °C, from a distance of 1.5 m, which meets the criteria for thermal imaging tests.

**Figure 2:**
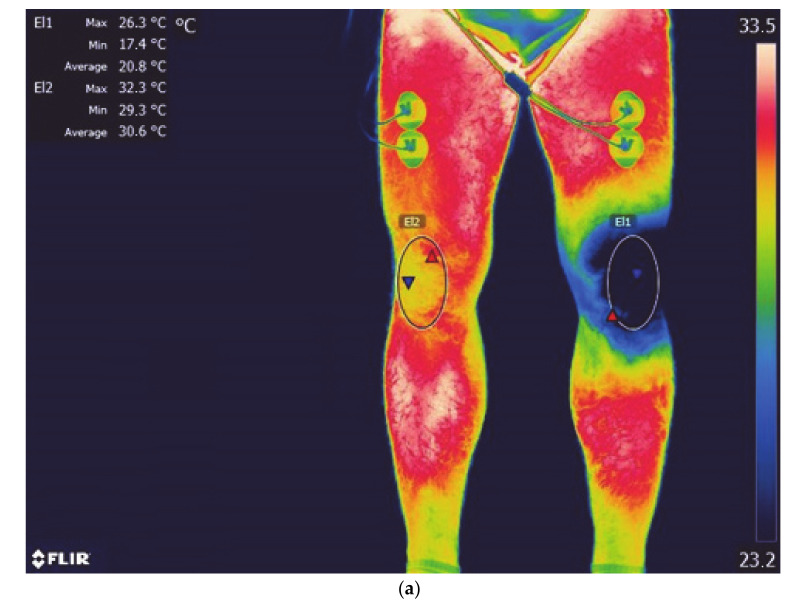

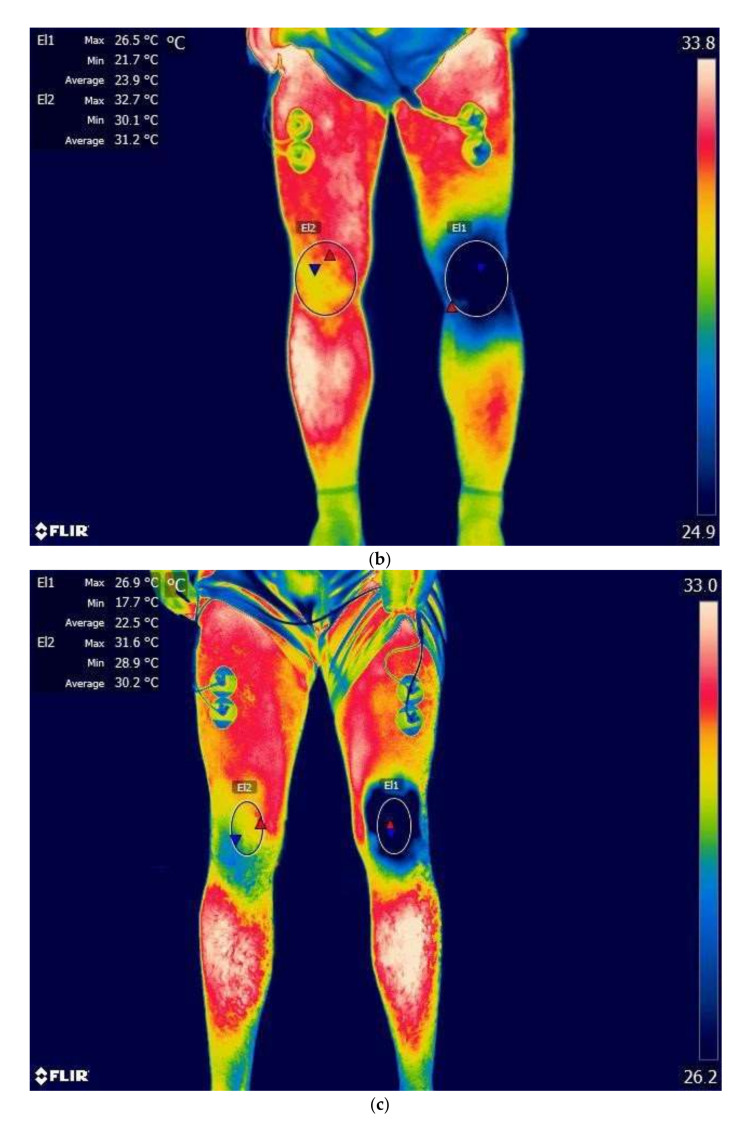
Lower limb thermogram with marked areas (El1- ROI_E_, El2- ROI_NE_); (**a**) liquid nitrogen vapors; (**b**) cold air; (**c**) ice bag.

The measurements were performed with a FLIR A655sc digital infrared camera (Flir Systems Inc., Wilsonville, OR, USA) with noise-equivalent temperature difference (NETD) < 0.05 °C, focal plane sensor array size of 640 × 480, and measurement uncertainty of ±2% of the overall operational temperature range. It streams full-frame 16-bit data at 50 Hz, or up to 200 Hz with windowing, for high-speed processes. Its standard temperature range is −40 °C to 150 °C (−40 °F to 302 °F) [53]. The emissivity was set at 0.98. The analysis used the mean temperature value (T_mean_) from a region of interest (ROI) using the original FLIR software (FLIR Tools+) from the areas of the anterior surface of both lower limbs: ROI_E_—the area of the knee joint exposed to cooling agents (left lower limb), ROI_NE_—the area of the knee joint of the contralateral (unexposed, right) limb (Figure 2a–c).

### 2.4. Conditions and Procedures for the Application of Cooling Agents.

The application of LNV was performed from a distance of 10–15 cm of the nozzle from the skin, in circular movements around the patella, using a KRIOMEDPOL KRIOPOL R11 (Kriomedpol, Stare Babice, Poland) apparatus. In the apparatus used, vapors are created by heating liquid nitrogen with a heater in the device’s cylinder (gas stream temperature at the nozzle outlet specified by the manufacturer −160 °C, liquid nitrogen consumption 0.15 kg/min of continuous operation).

The application of CA was carried out with a reduction in the distance to 2–5 cm from the skin surface and the use of a ZIMMER CRYO 6 apparatus (Zimmer MedizinSysteme GmbH, Hamburg, Germany, 2013). In the ZIMMER CRYO 6 apparatus, CA is formed as a result of cooling the air sucked in from the environment, in a closed refrigeration circuit system (the temperature specified by the manufacturer at the nozzle outlet is approx. −30 °C). A blow-in intensity of 9 was used (1000 L/min, nozzle diameter 15 mm).

The application of ice to the knee joint was made with IB (MUELLER, 23 cm), applying it directly to the skin surface of the knee joint for 3 min. IB was filled with ice cubes, with an average weight of 1100 g.

The scope of duties during the study was divided between 2 appropriately trained researchers: one person was responsible for pre-treatment procedures and the performance of the procedure, while the other was responsible for taking thermographic images and their subsequent processing. The tests were carried out in the morning (8:00–11:00 a.m.) and in accordance with the standard laboratory protocol of the measuring and testing equipment used.

None of the participants in this study reported a negative reaction to the applied cooling agents.

### 2.5. Statistical Analysis

At the stage of study planning, a sample size calculation was made using G*Power 3.1.9.7 software (https://www.psychologie.hhu.de/arbeitsgruppen/allgemeine-psychologie-und-arbeitspsychologie/gpower.html): for ANOVA, repeated measures, within factors (input parameters: effect size f = 0.5940885, α error probability = 0.05, power (1-β error probability) = 0.95, output parameters: total sample size = 6, actual power = 0.9999969), t tests, difference between two independent means—two groups (input parameters: effect size d =1.9587758, α error probability = 0.05, power (1-β error probability) = 0.95, output parameters: sample size group 1 = 7, sample size group 2 = 7, total sample size = 14, actual power = 0.9642649), t tests, difference between two dependent means—matched pairs (effect size = 1.0406118, α error probability = 0.05, power (1-β error probability) = 0.8; output parameters: total sample size = 8, actual power = 0.8410043). Based on the results of a priori analysis obtained, the minimum group size was established to be 12 individuals. The statistical analysis of the results was performed using Statistica 13.3 software (Statistica PL, StatSoft, Kraków, Poland). The data distribution was examined using the Shapiro–Wilk test and a data scatter plot. In the case of a normal distribution of data, the characteristics of the examined variables was presented in the form of arithmetic means and standard deviation, while for the variables with a distribution deviating from the normal, in the form of median and minimum and maximum values. To test the significance of differences between pairs of independent variables with a normal distribution, the Student’s *t*-test was used. The one-way ANOVA to compare the means of many variables (between measurements at 24 assessed time points, between three assessed cooling agents in these same time points). If significant differences were found in the univariate analysis of variance, the analysis was supplemented with Tukey’s HSD post hoc tests. The *p* value < 0.05 was considered statistically significant. Additionally, an ANOVA was performed for repeated measurements to assess the effect of the cooling agent on the temperature ROI_E_ and ROI_NE_ change over time. The assumption of spherical variance was tested with the Mauchly test. As the test results revealed a significant impairment of sphericity, a multivariate model was used in further analysis of variance for repeated measurements.

## 3. Results

The study group, whose results were included in the final analysis, consisted of 11 women (mean age: 25.33 years; mean BMI: 23.91 kg/m^2^) and 12 men (mean age: 28 years; mean BMI: 25.05 kg/m^2^). All the subjects were characterized by the correct values of the weight–growth index, the values of all assessed hemodynamic indices and physical activity satisfactory according to IPAQ (>600 MET).

The value of supragenual skinfold was measured for both limbs to determine the mean thickness of the insulating adipose tissue. There was no difference in the thickness of this skinfold, both the side-related and the inter-gender one.

The analysis of the baseline T_mean_ values for the areas of the knee joint, assessed three times in each subject (on consecutive days of the study), showed no side-related (right vs. left), inter-gender differentiation and, at the same time, high repeatability between measurements. The mean skin surface temperature for the right and left knee was 29.31 ± 1.28 °C and 29.18 ± 1.11 °C, respectively (Table 1).

Due to the lack of differences between the sexes in the indicated parameters characterizing the subjects, it was decided to analyze the entire group without dividing it by gender.

The assessment of the thermal response was interpreted based on the analysis of changes in the skin surface temperature of the stimulated (left) and non-stimulated (right) knee joint area during a 90-min observation period.

**Figure 3:**
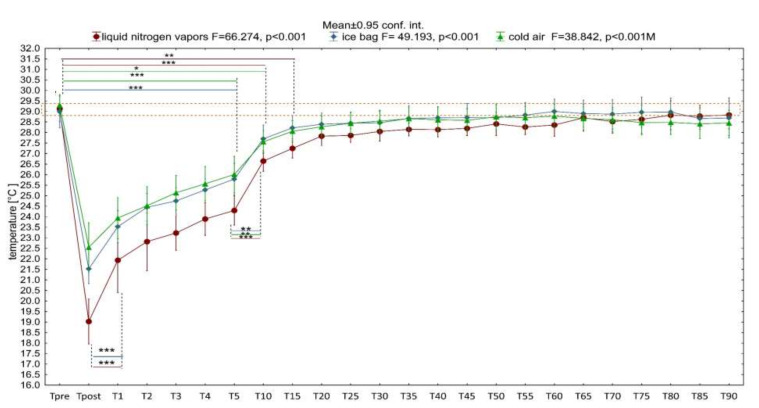
Change in the mean surface temperature of the knee joint after a 3-min application of three cooling agents. Legend: Data are expressed as mean with standard deviation. The dashed orange line represents the average ROI temperature before exposure. Two-way repeated measures ANOVA, F—ANOVA value, *p*—test probability level, test probability level determination for Tukey’s HSD post hoc tests: * *p* <0.05, ** *p* <0.01, *** *p* <0.001.

Immediately after a 3-min exposure (T_post_), regardless of the type of C_Ag_ used, the skin temperature of the stimulated knee joint decreased significantly: in the case of LNV, it decreased by 10.11 ± 0.91 °C; T_mean_ = 19.02 ± 2.07 °C, after IB application it decreased by 7.59 ± 0.14 °C; T_mean_ = 21.52 ± 1.36 °C, and after CA application it decreased by 6.76 ± 1.3 °C; T_mean_ = 22.55 ± 2.28 °C (Figure 3). The size of the T_mean_ change in the case of LNV stimulation was significantly greater than in the remaining C_Ag_.

At the following measurement points, starting from the first minute after the end of exposure (T_1_), the temperature of the cooled area gradually increased (Figure 3), still remaining significantly below the baseline value up to 5 min for IB application (*p* < 0.001), up to 10 min after CA application (*p* < 0.05) and up to 15 min after LNV application (*p* < 0.01).

**Figure 4:**
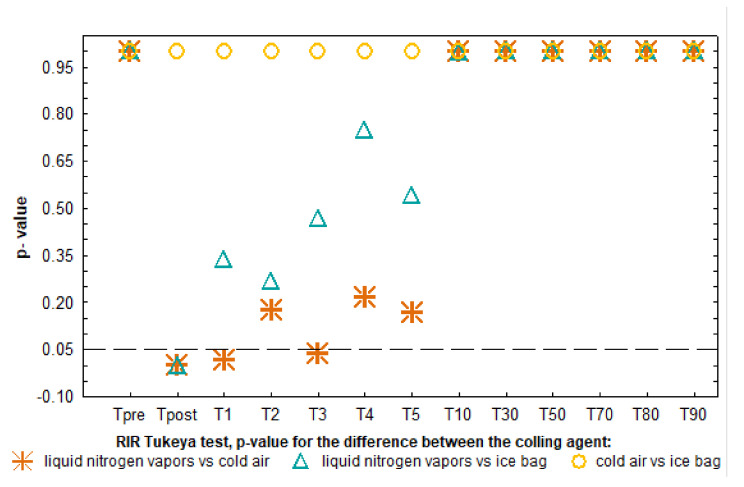
Analysis of changes in the significance of differences over time between surface temperatures after the application of cooling agents.

The analysis of the dynamics of temperature changes during the 90-min observation (Figure 3) showed a rapid significant increase in temperature in the first minute from the end of the stimulus action (LNV and IB), then a slow, nonsignificant increase in temperature in the following minutes between 1 and 5 min and a significant increase in temperature between 5 and 10 min after exposure (regardless of the C_Ag_) (Figure 3). When analyzing in detail the differentiation of responses to C_Ag_, it was shown that the T_mean_ of the knee joint area immediately after LNV stimulation was significantly lower than after CA and IB application (*p* < 0.05) and at 1 and 3 min only in relation to CA (*p* < 0.05). Regardless of the C_Ag_ used from 3 min after application, the recorded temperatures did not show any significant differences. At the same time, throughout the 90-min observation period, the surface temperatures after IB and CA application did not differ significantly (Figure 4).

**Figure 5:**
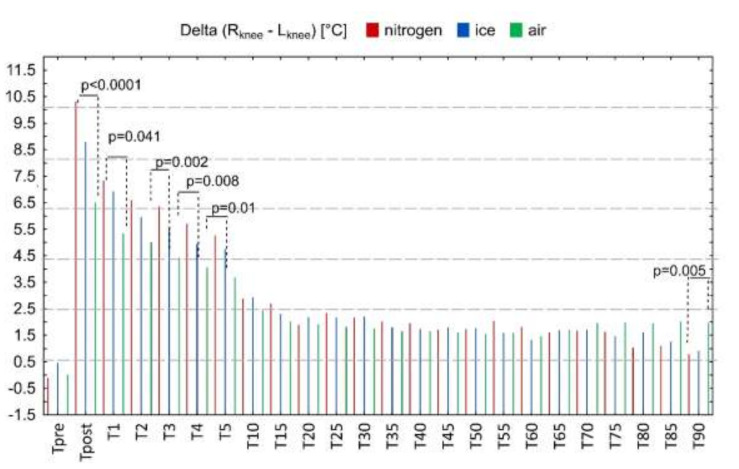
Change in the value of the difference in temperature between the areas of the right (unstimulated) and left (stimulated) knee joint during 90-min observation. Legend: *p*—the level of test probabilities for one-way ANOVA for the differences between liquid nitrogen vapors, cold air, ice bag at the estimated time point. Data are expressed as mean.

In the next stage, a comparative analysis of the temperature of the stimulated and non-stimulated knee joint was performed (Figure 5). The vertical lines show the mean values of the differences between the temperatures of contralateral knee joints (∆TR_knee_–L_knee_), which immediately after application were 10.31 ± 1.96 °C for LNV, 8.8 ± 1.47 °C for IB and 6.51 ± 2.34 °C for CA, respectively.

Between the direct measurement and the measurement made 5 min after the exposure, the ∆ (ROI_NE_–ROI_E_) value decreased, and its value was significantly different for the used cooling agents in the first, third, fourth, and fifth minutes (*p* < 0.05), being characterized by the highest value for LNV, the lower for IB and the lowest value for CA (Figure 5). From the 10th to the 85th minute of observation, the ∆(ROI_NE_–ROI_E_) value continued to decrease, but showed no significant inter-stimulus differentiation, remaining in the range from 1 to 3 °C. At the last observation point (T_90_), a significantly higher value of ∆(ROI_NE_– ROI_E_) was recorded after exposure to CA, compared to other C_Ag_.

**Figure 6:**
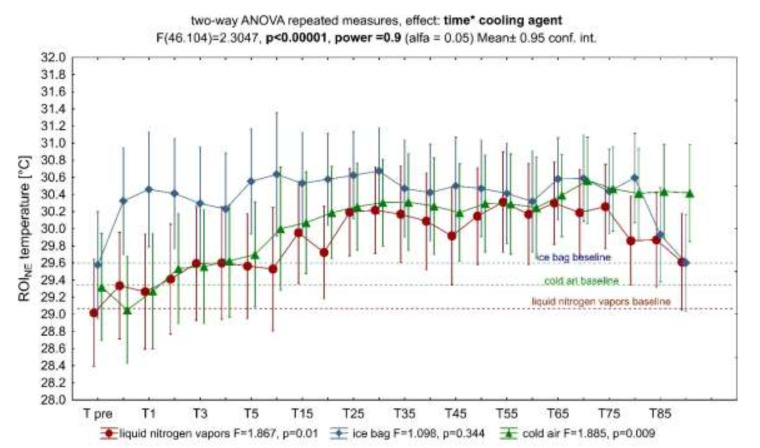
Characterization of the influence of exposure with cooling agents on the ROI_NE_ temperature during 90 min of observation. Legend: analysis of variance for repeated measurements, significance of the influence of time and cooling agent interactions on the ROI_NE_ temperature.

**Figure 7:**
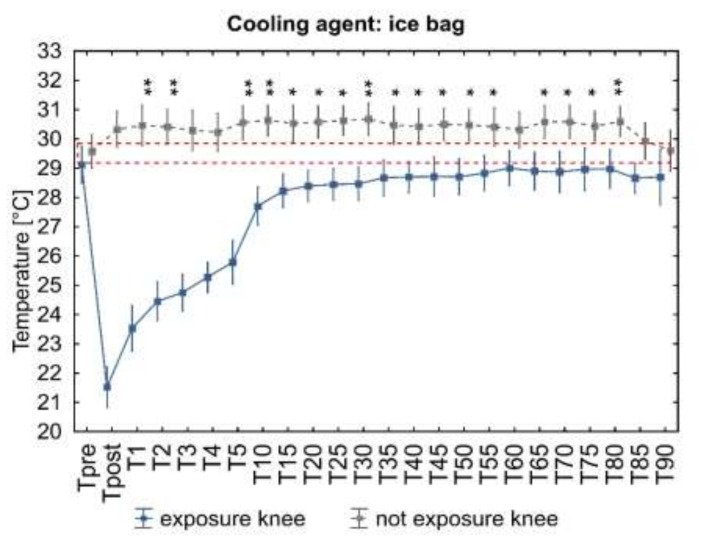

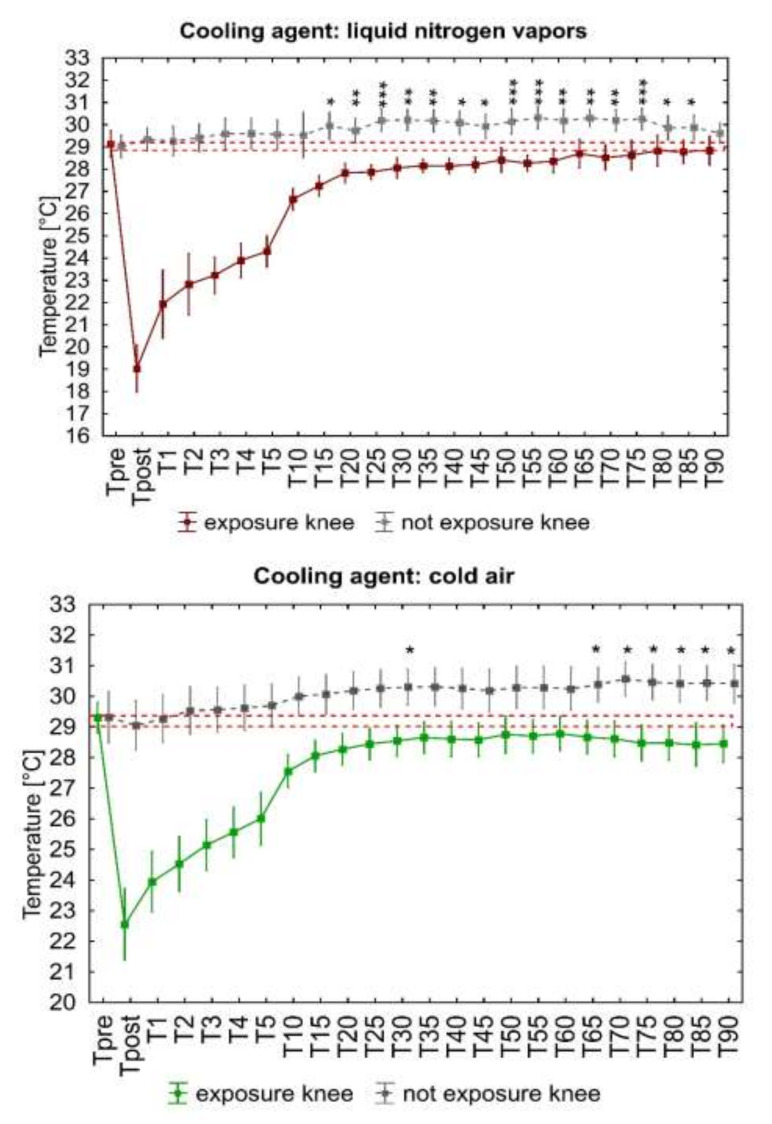
Analysis of ROI_NE_ and ROI_E_ surface temperature changes during 90 min of observation. Legend: Student t-test probability level between pre-exposure and post-exposure ROI_NE_ temperature (from T_post_ to T_90_): * *p* < 0.05, ** *p* = 0.001, *** *p* = 0.001. Data are expressed as mean with standard deviation; the dashed orange line represents the level of the mean output temperature for both ROI_NE_.

Exposure to the tested C_Ag_ caused an increase in the temperature of the contralateral area (unexposed limb), the dynamics of which depended on the cooling agent used (Figure 6). The analysis of variance for repeated measurements showed a significant influence interaction of time and the cooling agent on the change in ROI_NE_ temperature during the 90-min observation period (Figure 6). A significant increase in temperature was found, respectively, at 1, 2 and 5 to 55 min and from 60 to 80 min after IB application (Figure 7, ice bag), from 15 to 85 min after LNV application (Figure 7, liquid nitrogen vapors), and at 30 and from 65 to 90 min after the end of exposure to CA (Figure 7, cold air).

## 4. Discussion

The conducted research was aimed at evaluating the dynamics of changes in skin temperature of the knee joint as a result of a 3-min exposure to LC using three different C_Ag_. To the authors’ knowledge, this is the first study in which a comparative analysis of the thermal response to LVN, CA and IB stimuli was performed, standardizing exposure conditions and taking into account the response of the contralateral region. To date, methodological protocols have not been standardized depending on the choice of cooling agent, which seems necessary for their targeted clinical application. There are also few studies analyzing the thermal response to cooling based on which such standardization could be made. Among the available papers, the fewest studies concern refrigerant gases, as evidenced by the review paper by Joseph T. Costello et al. from 2012, in which out of 19 analyzed research works on thermal evaluation after LC treatments, only one concerned localized CA (and the rest concerned a cryotherapy cuff, crushed ice, cold water, after gel pack, cooling gel, ice massage, and frozen peas) [6]. Supplementing the data in the source material is extremely important in view of the increasing use of refrigerant gases (LNV, CA, carbon dioxide microcrystals) in clinical practice in LC treatments [16,20,37,51,54]. For this reason, in the presented study, the thermal response after LNV, CA and IB application was compared to determine their cooling potential based on the thermographic image.

In the presented study, LNV caused the largest and the longest-lasting temperature drop of the exposed area, which proves its greatest cooling potential among the assessed factors (Figure 3 and Figure 4). The cooling potential between IB and CA was comparable (no significant differences in the value of the stimulated surface temperature between these agents) and weaker compared to LNV (significantly higher values of the exposed area temperature) (Figure 3 and Figure 4). Regardless of the C_Ag_ used, already after the first minute from the end of the exposure, an increase in the temperature of the stimulated area was observed, with the highest significant increase in temperature between 5 and 10 min of observation. After this time, the temperature of the assessed area continued to increase, reaching values close to the baseline values. The difference between the skin temperature of the exposed area and the contralateral area was the highest between 1 and 5 min of observation, and its magnitude in this period was dependent on the C_Ag_ used. From the fifth minute of observation, the temperature difference between the contralateral limbs remained relatively constant, low, and its magnitude was similar for the cooling agents used (Figure 5). In the contralateral area, the skin temperature increased after the treatment, and the dynamics of this increase was dependent on the applied cooling agent, being the strongest after exposure to LNV, less after IB and the least after CA (Figure 6 and Figure 7).

The source data clearly confirm that the degree of tissue hypothermia depends on the C_Ag_ used, which is connected with a differences in the specific heat and the ability of the modalities to change phase from solid to liquid [6,51,55]. For example, the decrease in mean skin surface temperature after 20 min of applying IB compress, gel packs, frozen peas and water–alcohol mixture was 22 °C, 19 °C, 18 °C, 22 °C, respectively [55]. However, the use of carbon dioxide microcrystals for 2 min in a group of young, healthy individuals lowered the skin temperature by 25 °C [23]. In our own study, immediately after a 3-min exposure, a decrease in the temperature of the knee joint area was found, on average from 6.76 to 10.11 °C (Figure 3 and Figure 4), which is consistent with the literature data showing that the local application of various C_Ag_ (CA, gel packs, cryo cuff, cold water immersion, IB, frozen peas, carbon dioxide microcrystals, LNV) effectively lowers skin temperature by more than 5 °C in various areas of the body [6,23,51]. In the presented study, the temperature of the exposed area recorded immediately after LNV treatment was significantly lower than after exposure to CA and IB, which is partially consistent with the literature showing a higher cooling potential (lower skin temperature and intramuscular temperature after exposure) of liquefied gases (carbon dioxide microcrystals) than IB compress [23,51,54]. The value of CAg temperature is not the only important physical property influencing the cooling efficiency. The change of the physical state (from solid to liquid) during the exposure increases the cooling of the exposed tissue. This is confirmed by comparative studies showing greater cooling of the skin after simultaneous exposure to IB compared to gel pack and frozen peas [22,55]. In our own study, we showed a lower drop in temperature after IB, compared to the literature data [23,55], which was certainly affected by a five-fold shorter exposure time. When planning the research, we were aware that treatments involving compresses of cooling agents, including IB, are performed from 5 to even 20 min [6,23,55]; however, we wanted to directly compare the cooling potential of the assessed cooling agents, so we decided to standardize the exposure time. The duration of cryotherapeutic effect selected by us is based on clinical practice, in which it is recommended to use LNV and CA for no longer than 3 min [25,51]. The recommended 3-min duration of LC was applied to the hands of RA patients in clinical trials, obtaining significant reductions in the mean skin temperature of 11 °C and 6.3 °C, respectively, 1 min after LNV and CA exposure [51]. At the same time point, the temperature drop we demonstrated was somewhat smaller (LNV: 7.1 °C, and CA: 5.5 °C) (Figure 3). In other study, a 5-min local exposure of the knee joint to CA reduced skin temperature by 22.1 °C, and inside the joint by an average of 3.9 °C. [54]. The presented discrepancies in the value of the mean temperature drop after exposure between both our own data presented and the results from the literature in skin temperature after LC with CA may be a consequence of not only the extended exposure time (5 min), but also different levels of air cooling achieved in the apparatuses.

### 4.1. The Effect of Cooling on the Tissue

The analgesic effect of cryotherapy treatments is explained by a number of mechanisms, including decreased receptor sensitivity, decreased receptor firing rate, decreased nerve conduction velocity (NCV), reduced muscle spasm or as a counter irritant to pain. The threshold for optimal induction of analgesia in clinical conditions was determined, the value of which is from 10 to 13 °C of skin temperature, at which NCV decreases by 10–33% [1]. The analgesic effectiveness of LC treatments is therefore dependent on obtaining potentially long-term cooling of tissues without their thermal damage [34]. In the presented study, a 3-min exposure to the C_Ag_ tested did not lower the skin temperature to the level corresponding to the optimal analgesia threshold (Figure 3).

The physiological reaction of the surface blood vessels to locally lowering the temperature is their sudden narrowing, which is the result of an autonomic reflex reaction from the stimulation of skin thermoreceptors, direct stimulation of arterial smooth muscles and the reaction of precapillary sphincters due to the release of serotonin and bradykinin [1,6,34,38,51,56]. Still, during the action of cold (from 2 to 3 min), vasodilation (cold indocid vasodilation, CIVD) occurs, which intensifies rapidly after about 2 to 6 min after the end of the stimulus action, increasing the blood flow through the tissues several times (lasting up to 6 h). In the thermographic image, the post-exposure skin temperature increases with time, as a consequence of both increased blood circulation and as a result of heat absorption by surface tissues from deeper tissues (Figure 3) [1,23,51,57]. Post-exposure increased hyperemia often constitutes the overriding therapeutic goal, as it creates conditions for the improvement of metabolism, elimination of metabolic products such as lactate or histamine, and an increase in bradykinin and angiotensin levels [38,58,59,60]. According to the literature, the induction of reflex vasoconstriction and next CIVD in the skin in response to local cooling requires a drop in skin temperature by 5 to 10 °C [61]. In the presented research, a 3-min application of both cooling gases (CA, LNV) and IB allowed one to obtain an appropriate decrease in skin temperature of the exposed area. (Figure 3).

Muscle cooling produces physiological effects manifested by reduced activity of the muscle (neuromuscular) spindle, reduced nerve conduction velocity and receptor potential release, as well as reduced ATP hydrolysis and impaired release and uptake of calcium in the muscle [1]. Additionally, according to the van’t Hoff rule, lowering the tissue temperature by 10 °C will result in a two-fold slowing down of the chemical reactions taking place in it. This phenomenon can be used in the treatment of acute injuries. Slow cell metabolism reduces the risk of secondary enzymatic and ischemic damage, thereby reducing the overall extent of tissue damage and the inflammatory process. However, there are no human studies on this hypothesis, which makes it difficult to accurately determine the threshold value necessary to obtain the skin temperature [1]. Due to the insufficient and short-term level of the obtained skin cooling, the possibility of obtaining the above-mentioned effects as a consequence of a 3-min exposure of the knee joint to LNV, CA and IB (Figure 3) is considered unlikely. In the present study, the temperature of the stimulated area returned to the baseline values 10 (IB), 15 (CA), 20 (LNV) minutes after the end of the LC treatment (Figure 3).

In the presented study, exposure to cooling agents caused ROI to cool down immediately after the treatment, followed by a systematic increase in skin temperature (Figure 3). Up to 10–15 min after exposure, the most rapid post-treatment temperature increase was observed, which is consistent with the source data [23,51,54,55]. Although the used cooling agents were characterized by different temperatures, the dynamics of skin temperature increase after exposure was comparable (Figure 3). According to the source data, after applying IB, the temperature of the exposed area returns slowly and smoothly, while after exposure to refrigerant gases, it returns sharply and much faster [23,51,55]. In our opinion, the difference in the dynamics of the observed thermal response in the literature may be due to the duration of the procedure. IB in the cited sources was used for a long time, causing tissue cooling, a significant decrease in tissue metabolism with a slow increase in temperature after the treatment [23,55]. In the presented study, the purpose of using cooling agents (also IB) in a short and uniform time of their effect was to compared the possibility of inducing the stimulating effect of cold (Figure 3), which is typical for the short-term use of refrigerant gases (CA, carbon dioxide microcrystals, LNV) [23,51]. It seems obvious that cooling agents with a higher cooling potential, disturbing the thermal homeostasis of the organism more rapidly, may induce a more intense thermoregulatory response of the organism [23,39]. Our observations show, however, that the use of a cooling agent with a higher temperature, but different physical and thermodynamic properties, may be sufficient to obtain the appropriate cooling potential (IB vs. LNV) and in a short action time may be suitable for starting a rapid thermoregulatory response (Figure 3 and Figure 4). Studies evaluating the relationship between skin temperature and that of deep tissues (muscles) show that when skin temperature increases after the end of exposure to a cooling agent, the muscle temperature decreases. The described phenomenon is consistent with the second law of thermodynamics (heat transfer is always unidirectional from high to low values), resulting in the absorption of heat by surface tissues from deeper tissues. That is why the maximum effect of lowering muscle temperature is observed after the end of exposure, while the skin temperature increases [54,62]. The knowledge of the skin temperature change after the finished exposure allows for inference about the temperature change in deeper tissues, which was presented in the study by Hardaker et al. from 2007 [62]. A strong negative correlation was demonstrated between intramuscular temperature and skin temperature, in line with the assumption that the amount of heat that an object can hold is directly proportional to its volume, and therefore the muscle temperature can be calculated by means of dispersion [6,62]. In the presented context, it is worth following the change in skin temperature after LC in order to estimate the cooling of deeper tissues (in the absence of direct assessment), which is allowed by thermographic tests.

### 4.2. A Contralateral Reaction

A consensual reaction is an identical response of the organism on the side contralateral to the area of stimulation. According to the source data, it also occurs in relation to vessels after LC application [16,51,63,64]. The cited sources describe contralateral reactions consisting in an initial decrease and then an increase in the temperature of the unexposed area, similar in its time course to the temperature change in the exposed area, after LC treatments with LNV, CA and carbon dioxide microcrystals [16,23,51]. In the authors’ own research, a significant increase in the skin temperature of the knee joint in the unexposed limb compared to the exposed limb was observed. The dynamics and course of said temperature increase were dependent on the C_Ag_ used, being the strongest after application of LNV, less after IB and least after CA (Figure 5 and Figure 6). It can therefore be concluded that the strength of the contralateral reaction of the vessels depends on the cooling potential of the cooling agent used in the exposed area, which is also confirmed by the results of the source data [16,23,51].

Our study did not confirm an initial temperature drop in the contralateral region, showing only an increase in temperature (Figure 6). Perhaps, in order to obtain a full consensual reaction, it is necessary to cool the skin more, as evidenced by such a reaction after using hyperbaric gas cryotherapy (lowering the temperature of the exposed skin from 32.5° ± 0.5 °C to 7.3° ± 0.8 °C) and in the same subjects, there was no consensual reaction after the IB (lowering the temperature of the exposed skin from 32.5° 0.6 °C to 13.9° ± 0.7 °C) [23]. On the other hand, in other subjects, a consensual reaction was obtained on the opposite side with similar cooling (LNV decrease in the temperature of the skin surface of the exposed hand from 28.9 ± 1.8 °C to 17.9 ± 2.2 °C, and CA from 29.4 ± 2.4 °C to 23.1 ± 2.2 °C) as in our study, which may indicate other determining factors [51]. We assume that the application area was crucial. The hands are characterized by a thinner layer of subcutaneous fat and a large number of shallowly located blood vessels compared to the abdominal area of the knee joint. The second decisive factor may be the application time in the mentioned studies are significantly shorter for LNV and CA compared to IB [23,51]. CIVD responses have been shown to be more pronounced when the baseline body and skin temperature is high and suppressed when temperature is low, compared to normothermia [65]. It is possible that short term exposure to extremely low temperatures is necessary to induce a full consensual response. Such exposure will be sufficient to stimulate blood vessels’ reaction without deep and long-term cooling of the tissues inhibiting CIVD. The inclusion of a contralateral reaction seems to be of great importance in clinical practice, in some circumstances as an additional therapeutic benefit, in others a potential risk of inducing adverse reactions. The contralateral reaction is believed to be a systemic response, with the involvement of the autonomic nervous system [23,63], or a peripheral spinal-mediated peripheral response without being affected by the central thermoregulation system [66].

## 5. Conclusions

The three-minute exposure of the knee joint to LNV, CA and IB significantly disturbs regional thermal hemostasis. The strongest response of the body is seen after exposure to LNV and the weakest after exposure to CA. The thermoregulatory response is general in nature: it covers both the exposed and the unexposed limb, which is reflected in changes in the surface temperature of both areas. Regardless of the cooling agent used, a characteristic course of the systemic reaction to the 3-min local stimulating effect of low temperatures is observed. The strongest response of the organism occurs immediately after the application of the cooling agent and for the first minute after its application, then it loses its intensity and increases again between 5 and 10 min of observation. The reaction of the unexposed, contralateral ROI is different for individual cooling agents in terms of the intensity of the temperature increase and the time points of their occurrence.

### Limitations

Due to the relatively small study group, we realize that this is an important factor in the limitations of the study. In the course of this research, several aspects emerged that should be developed in further studies. A comparative assessment of the thermal effect over time would be highly reasonable, taking into account the planned stimulating effect of the application of cold depending on the cooling effect, including the duration and temperature of exposure. It is also reasonable to pay attention to the possibility of inter-gender differentiation of response.

## Figures and Tables

**Table 1:** Characteristics of the study group taking into account gender.

	Study Group		Women (n = 11)		Men (n = 12)		Women vs. Men
N = 23 (W = 11, M = 12)	mean/ * median	±SD/ * (min–max)	mean/ * median	±SD/ * (min–max)	mean/ * median	±SD/ * (min–max)	*p*-value test *t*/ * U-Mann–Whitney
Age (years)	26.67	4.56	25.33	4.87	28.00	4.06	0.225
Body hight (m)	1.72	0.08	1.66	0.06	1.77	0.06	0.002
Body weight (kg)	72.82	14.27	66.06	16.05	78.82	9.78	0.063
BMI (kg/m^2^)	22.51	1.80	21.91	1.12	24.05	1.29	0.557
SF_R_ (mm)	17.12	5.29	18.90	2.22	14.71	5.94	0.106
SF_L_ (mm)	16.81	4.82	18.96	2.88	15.29	6.68	0.206
ABI_R_	1.09	0.05	1.06	0.08	1.11	0.04	0.519
ABI_L_	1.10	0.06	1.09	0.07	1.12	0.04	0.669
HR	73.22	3.86	75.70	1.34	72.74	4.28	0.299
SBP (mm/Hg)	125.70	3.16	126.11	4.04	125.30	4.73	0.288
DBP (mm/Hg)	81.43	2.33	82.30	2.19	83.56	1.16	0.228
IPAQ (MET) *	1466.5	(693–8238)	1466.5	(698–6279)	5278	(693–8238)	0.862
T_pre_ ROI_E_ (°C)	29.18	1.11	29.24	0.91	29.13	1.27	0.731
T_pre_ ROI_NE_ (°C)	29.31	1.28	29.21	1.04	29.39	1.47	0.632

Legend: Data are expressed as mean with standard deviation or median (*) with minimum and maximum. BMI—body mass index, SF_R_—skinfold right lower limb, SF_L_—skinfold left lower limb, ABI_R_—ankle–brachial index right lower limb, ABI_L_—ankle–brachial index left lower limb, HR—heart rate, SBP—blood pressure systole, DBP—blood pressure diastole, IPAQ—International Physical Activity Questionnaire, MET—metabolic equivalent of work, T_pre_: ROI_E_—mean temperature of the area of the knee joint before exposed to cooling agents (left lower limb), ROI_NE_—mean temperature of the area of the knee joint of the contralateral, before exposure (unexposed, right).

## Data Availability

Not applicable.

## References

[1] Bleakley C.M., Hopkins J.T. (2010). Is it possible to achieve optimal levels of tissue cooling in cryotherapy?. Phys. Ther. Rev..

[2] Hermann J. (2009). Kryotherapie. Z. Rheumatol..

[3] Cholewka A., Stanek A., Sieroń A., Drzazga Z. (2012). Thermography study of skin response due to whole-body cryotherapy. Ski. Res. Technol..

[4] Bouzigon R., Grappe F., Ravier G., Dugue B. (2016). Whole- and partial-body cryostimulation/cryotherapy: Current technologies and practical applications. J. Therm. Biol..

[5] Swenson C., Swärd L., Karlsson J. (1996). Cryotherapy in sports medicine. Scand. J. Med. Sci. Sport..

[6] Costello J.T., McInerney C.D., Bleakley C.M., Selfe J., Donnelly A.E. (2012). The use of thermal imaging in assessing skin temperature following cryotherapy: A review. J. Therm. Biol..

[7] Bayindir S.K., Çürük G.N., Oguzhan A. (2017). Effect of Ice Bag Application to Femoral Region on Pain in Patients Undergoing Percutaneous Coronary Intervention. Pain Res. Manag..

[8] Guillot X., Tordi N., Mourot L., Demougeot C., Dugué B., Prati C., Wendling D. (2014). Cryotherapy in inflammatory rheumatic diseases: A systematic review. Expert Rev. Clin. Immunol..

[9] Peres D., Sagawa Y., Dugué B., Domenech S.C., Tordi N., Prati C. (2017). The practice of physical activity and cryotherapy in rheumatoid arthritis: Systematic review. Eur. J. Phys. Rehabil. Med..

[10] Bleakley C., McDonough S., Gardner E., Baxter G.D., Hopkins J.T., Davison G.W. (2012). Cold-water immersion (cryotherapy) for preventing and treating muscle soreness after exercise. Cochrane Database Syst. Rev..

[11] Banfi G., Lombardi G., Colombini A., Melegati G. (2010). Whole-body cryotherapy in athletes. Sport. Med..

[12] Hohenauer E., Costello J.T., Stoop R., Küng U.M., Clarys P., Deliens T., Clijsen R. (2018). Cold-water or partial-body cryotherapy? Comparison of physiological responses and recovery following muscle damage. Scand. J. Med. Sci. Sport..

[13] Pawik M., Kowalska J., Rymaszewska J. (2019). The effectiveness of whole-body cryotherapy and physical exercises on the psychological well-being of patients with multiple sclerosis: A comparative analysis. Adv. Clin. Exp. Med..

[14] Giemza C., Matczak-Giemza M., Ostrowska B., Bieć E., Doliński M. (2014). Effect of cryotherapy on the lumbar spine in elderly men with back pain. Aging Male.

[15] Nadler S.F., Weingand K., Kruse R.J. (2004). The Physiologic Basis and Clinical Applications of Cryotherapy and Thermotherapy for the Pain Practitioner. Pain Physician.

[16] Boerner E., Podbielska H. (2018). Application of thermal imaging to assess the superficial skin temperature distribution after local cryotherapy and ultrasound. J. Therm. Anal. Calorim..

[17] De Nardi M., Silvani S., Ruggeri P., Luzi L., La Torre A., Codella R. (2019). Local cryostimulation acutely preserves maximum isometric handgrip strength following fatigue in young women. Cryobiology.

[18] Guillot X., Tordi N., Prati C., Verhoeven F., Pazart L., Wendling D. (2017). Cryotherapy decreases synovial Doppler activity and pain in knee arthritis: A randomized-controlled trial. Jt. Bone Spine.

[19] Guillot X., Tordi N., Laheurte C., Pazart L., Prati C., Saas P., Wendling D. (2019). Local ice cryotherapy decreases synovial interleukin 6, interleukin 1β, vascular endothelial growth factor, prostaglandin-E2, and nuclear factor kappa B p65 in human knee arthritis: A controlled study. Arthritis Res. Ther..

[20] Kennet J., Hardaker N., Hobbs S., Selfe J. (2007). Cooling efficiency of 4 common cryotherapeutic agents. J. Athl. Train..

[21] Ni S.H., Jiang W.T., Guo L., Jin Y.H., Jiang T.L., Zhao Y., Zhao J. (2015). Cryotherapy on postoperative rehabilitation of joint arthroplasty. Knee Surg. Sport. Traumatol. Arthrosc..

[22] Breslin M., Lam P., Murrell G.A.C. (2015). Acute effects of cold therapy on knee skin surface temperature: Gel pack versus ice bag. BMJ Open Sport Exerc. Med..

[23] Mourot L., Cluzeau C., Regnard J. (2007). Hyperbaric Gaseous Cryotherapy: Effects on Skin Temperature and Systemic Vasoconstriction. Arch. Phys. Med. Rehabil..

[24] Guilhem G., Hug F., Couturier A., Regnault S., Bournat L., Filliard J.R., Dorel S. (2013). Effects of air-pulsed cryotherapy on neuromuscular recovery subsequent to exercise-induced muscle damage. Am. J. Sports Med..

[25] Happe S., Evers S., Thiedemann C., Bunten S., Siegert R. (2016). Whole body and local cryotherapy in restless legs syndrome: A randomized, single-blind, controlled parallel group pilot study. J. Neurol. Sci..

[26] Song J.W., Kim H.H., Park E.J., Ahn J.H., Yoon E., Lampotang S., Gravenstein N., Choi S.C. (2018). Pre-emptive ice cube cryotherapy for reducing pain from local anaesthetic injections for simple lacerations: A randomised controlled trial. Emerg. Med. J..

[27] Lan L., Qian X.L., Lian Z.W., Lin Y.B. (2018). Local body cooling to improve sleep quality and thermal comfort in a hot environment. Indoor Air.

[28] Bender A.L., Kramer E.E., Brucker J.B., Demchak T.J., Cordova M.L., Stone M.B. (2005). Local ice-bag application and triceps surae muscle temperature during treadmill walking. J. Athl. Train..

[29] Hohenauer E., Deliens T., Clarys P., Clijsen R. (2019). Perfusion of the skin’s microcirculation after cold-water immersion (10 °C) and partial-body cryotherapy (−135 °C). Ski. Res. Technol..

[30] Diong J., Kamper S.J. (2014). Cold water immersion (cryotherapy) for preventing muscle soreness after exercise. Br. J. Sports Med..

[31] Khoshnevis S., Craik N.K., Diller K.R. (2015). Cold-induced vasoconstriction may persist long after cooling ends: An evaluation of multiple cryotherapy units. Knee Surg. Sport. Traumatol. Arthrosc..

[32] Tassignon B., Serrien B., de Pauw K., Baeyens J.P., Meeusen R. (2018). Continuous knee cooling affects functional hop performance -A randomized controlled trial. J. Sport. Sci. Med..

[33] Khoshnevis S., Craik N.K., Brothers R.M., Diller K.R. (2016). Cryotherapy-Induced Persistent Vasoconstriction After Cutaneous Cooling: Hysteresis Between Skin Temperature and Blood Perfusion. J. Biomech. Eng..

[34] Johnson J.M., Kellogg D.L. (2010). Local thermal control of the human cutaneous circulation. J. Appl. Physiol..

[35] Bagavathiappan S., Saravanan T., Philip J., Jayakumar T., Raj B., Karunanithi R., Panicker T.M.R., Korath M., Jagadeesan K. (2009). Infrared thermal imaging for detection of peripheral vascular disorders. J. Med. Phys..

[36] Bauer J., Dereń E. (2014). Standaryzacja badań termograficznych w medycynie i fizykoterapii. Acta Bio-Opt. Inform. Medica Inżynieria Biomed..

[37] Adamczyk J.G., Krasowska I., Boguszewski D., Reaburn P. (2016). The use of thermal imaging to assess the effectiveness of ice massage and cold-water immersion as methods for supporting post-exercise recovery. J. Therm. Biol..

[38] Christmas K.M., Patik J.C., Khoshnevis S., Diller K.R., Brothers R.M. (2018). Pronounced and sustained cutaneous vasoconstriction during and following cyrotherapy treatment: Role of neurotransmitters released from sympathetic nerves. Microvasc. Res..

[39] Chesterton L.S., Foster N.E., Ross L. (2002). Skin temperature response to cryotherapy. Arch. Phys. Med. Rehabil..

[40] Moreira D.G., Costello J.T., Brito C.J., Adamczyk J.G., Ammer K., Bach A.J.E., Costa C.M.A., Eglin C., Fernandes A.A., Fernández-Cuevas I. (2017). Thermographic imaging in sports and exercise medicine: A Delphi study and consensus statement on the measurement of human skin temperature. J. Therm. Biol..

[41] Cholewka A., Drzazga Z., Michnik A., Sieron A., Wisniowska B. (2004). Temperature Effects of Whole Body Cryotherapy Determined by Thermography. Thermol. Int..

[42] Cholewka A., Drzazga Z., Sieroń A. (2006). Monitoring of whole body cryotherapy effects by thermal imaging: Preliminary report. Phys. Med..

[43] Karki A., Karppi P., Ekberg J., Selfe J. (2004). A thermographic investigation of skin temperature changes in response to a thermal washout of the knee in healthy young adults. Thermol. Int..

[44] Costello J.T., Culligan K., Selfe J., Donnelly A.E. (2012). Muscle, skin and core temperature after −110 °C cold air and 8 °C water treatment. PLoS ONE.

[45] Costello J.T., Donnelly A.E., Karki A., Selfe J. (2014). Effects of whole body cryotherapy and cold water immersion on knee skin temperature. Int. J. Sports Med..

[46] Polidori G., Marreiro A., Pron H., Lestriez P., Boyer F.C., Quinart H., Tourbah A., Taïar R. (2016). Theoretical modeling of time-dependent skin temperature and heat losses during whole-body cryotherapy: A pilot study. Med. Hypotheses.

[47] Zalewski P., Łukowicz M., Zając A., Weber-Zimmermann M., Ciechanowska K. (2007). Porównawcza ocena termowizyjna skutków terapii zimnem z zastosowaniem różnych czynników chłodzących. Acta Bio-Opt. Inform. Med. Inżynieria Biomed..

[48] Wilk M., Trąbka R., Śliwiński Z. (2008). Zmiany obrazu termowizyjnego okolicy stawu kolanowego u pacjentów poddanych krioterapii miejscowej w zależności od stosowanego programu fizjoterapii. Fizjoterapia Pol..

[49] Krukowska J., Dalewski M., Czernicki J. (2014). Ocena skuteczności krioterapii miejscowej u osób ze spastycznością po udarze mózgu. Wiad. Lek..

[50] Szczepańska P., Skalska-Izdebska R., Goraj-Szczypiorowska B., Kurach A., Pałka T. (2012). Wpływ Krioterapii Miejscowej w Leczeniu Pacjentów z Gonartrozą.

[51] Korman P., Straburzyńska-Lupa A., Romanowski W., Trafarski A. (2012). Temperature changes in rheumatoid hand treated with nitrogen vapors and cold air. Rheumatol. Int..

[52] Elżbieta Biernat R.S.A.K.G. (PDF) International Physical Activity Questionnaire (IPAQ)-Polish Version. https://www.researchgate.net/publication/234833002_International_Physical_Activity_Questionnaire_IPAQ_-_Polish_version.

[53] FLIR A655sc High-Resolution Science Grade LWIR Camera | FLIR Systems. https://www.flir.com/products/a655sc/.

[54] Kim Y.H., Baek S.S., Choi K.S., Lee S.G., Park S.B. (2002). The effect of cold air application on intra-articular and skin temperature in the knee. Yonsei Med. J..

[55] Kanlayanaphotporn R., Janwantanakul P. (2005). Comparison of skin surface temperature during the application of various cryotherapy modalities. Arch. Phys. Med. Rehabil..

[56] Herrera E., Sandoval M.C., Camargo D.M., Salvini T.F. (2010). Motor and sensory nerve conduction are affected differently by ice pack, ice massage, and cold water immersion. Phys. Ther..

[57] Daanen H.A.M. (2003). Finger cold-induced vasodilation: A review. Eur. J. Appl. Physiol..

[58] Sellwood K.L., Brukner P., Williams D., Nicol A., Hinman R. (2007). Ice-water immersion and delayed-onset muscle soreness: A randomised controlled trial. Br. J. Sports Med..

[59] Vaile J., Halson S., Gill N., Dawson B. (2008). Effect of hydrotherapy on recovery from fatigue. Int. J. Sports Med..

[60] Vaile J.M., Gill N.D., Blazevich A.J. (2007). The effect of contrast water therapy on symptoms of delayed onset muscle soreness. J. Strength Cond. Res..

[61] Johnson J.M., Yen T.C., Zhao K., Kosiba W.A. (2005). Sympathetic, sensory, and nonneuronal contributions to the cutaneous vasoconstrictor response to local cooling. Am. J. Physiol.-Hear. Circ. Physiol..

[62] Hardaker N.J., Moss A.D., Richards J., Jarvis S., McEwan I., Selfe J. (2007). The relationship between skin surface temperature measured via non-contact thermal imaging and intra-muscular temperature of the rectus femoris muscle. Thermol. Int..

[63] Cooke J.P., Creager M.A., Osmundson P.J., Shepherd J.T. (1990). Sex. Differences in Control. of Cutaneous Blood Flow. Circulation.

[64] Crossland S., Drysdale I. (2006). Investigation into the affectivity on cutaneous blood perfusion of different application time lengths of cold hydrotherapy. Int. J. Osteopath. Med..

[65] Alba B.K., Castellani J.W., Charkoudian N. (2019). Cold-induced cutaneous vasoconstriction in humans: Function, dysfunction and the distinctly counterproductive. Exp. Physiol..

[66] Pulst S.M., Haller P. (1981). Thermographic assessment of impaired sympathetic function in peripheral nerve injuries. J. Neurol..

